# Mitochondrial genome of a leaf-mining beetle *Prionispa champaka* Maulik (Coleoptera: Chrysomelidae: Cassidinae)

**DOI:** 10.1080/23802359.2017.1413318

**Published:** 2018-02-01

**Authors:** Jiasheng Xu, Chengqing Liao, Qingyun Guo, Chengpeng Long, Xiaohua Dai

**Affiliations:** a Leafminer Group, School of Life and Environmental Sciences, Gannan Normal University, Ganzhou, China;; b National Navel-Orange Engineering Research Center, Ganzhou, China

**Keywords:** Leaf-miner, Cassidinae, *Prionispa champaka*, mitochondrial genome, phylogenetic analysis

## Abstract

*Prionispa champaka* is a leaf-mining species which feeds on *Pollia* spp. and widely distributes in southern China. The complete mitogenomic sequence of *P. champaka* (Chrysomelidae: Cassidinae) was obtained and annotated, with a length of 20,494 bp. It was longer than those of other Chrysomelid species (not including Bruchinae) because of its much longer non-coding sequences. Gene arrangement and content of *P. champaka* was identical to the most common type in insects, and it was also biased toward AT (accounting for 78.4%). Phylogenetic analysis based on mitochondrial PCGs indicated that *P. champaka* was closely clustered with 5 other Cassidinae species, supporting the traditional morphological classification within Cassidinae.

The genus *Prionispa* Chapuis, 1875, which consists of 29 described species globally and 7 species in China, belongs to the tribe of Oncocephalini (Chrysomelidae: Cassidinae). It mainly occurs in the oriental tropics (Chen et al. 1986; Staines 2015). The larvae of *Prionispa* are leaf-miners and generally form irregular blotch mines (Liao et al. 2018). *P. champaka* feeds on *Pollia japonica* Thunb. and *P. siamensis* (Craib) Faden ex Hong (Commelinaceae) and widely distributes in southern China (Chen et al. 1986; Liao et al. 2018). In this study, the complete mitochondrial genome of *P. champaka* was obtained and annotated for the first time. The species was collected in 31 October 2016 from Anjishan mountain, Jiangxi Province, China (N 24.870, E 114.606). All studied specimens were deposited in the Leafminer Group, School of Life and Environmental Sciences, Gannan Normal University, Jiangxi, China.

The complete mitogenome of *P. champaka* (GenBank accession no. MG543679) was 20,494 bp in length. It was within the range from 14,257 bp to 26,613 bp which were reported in other sequenced Coleoptera genomes (Bae et al. 2004; Sayadi et al. 2017). However, it was longer than those of other Chrysomelid species (not including Bruchinae) (Nie and Yang 2014; Guo et al. 2017a, 2017b; Wang and Tang 2017), because of the longer non-coding sequences of *P. champaka*. With overall base composition of 41.80% As, 36. 60% Ts, 9.40% Gs and 12.20% Cs, the mitogenome was biased toward AT (accounting for 78.4%). The circular genome had 13 protein-coding genes, 22 tRNA genes, two rRNA genes and one AT-rich region. Gene arrangement and content of our species was identical to the most common type of the putative ancestor of insects (Cameron 2014). There exited 12 gene overlaps at gene junctions, but most of them were only 1–2 bp; the longest one (8 bp) existed between tRNA^Trp^ and tRNA^Cys^, and between tRNA^Tyr^ and COX1. For the 13 protein-coding genes, only four PCGs (NAD1, NAD4, NAD4l and NAD5) encoded on the L-strand, others on the H-strand; the shortest one is ATP8 gene (153 bp) and the longest one is NAD5 gene (1705 bp). All PCGs started with ATN (except for NAD1 which started with TTG), ended with T (NAD2, COX1, COX2, COX3, NAD3, NAD5 and COB), TAG (NAD1) and TAA (ATP8, ATP6, NAD4, NAD4l and NAD6). All of the 22 tRNAs had a typical cloverleaf secondary structure, except for tRNA^ser^(AGN) which had a shorter dihydrouridine. All tRNAs had normal lengths which ranging from 60 to 70 bp.

The concatenated nucleotide sequences of 13 PCGs from 13 species of four chrysomelid subfamilies were chosen for phylogenetic analysis, including five species of Cassidinae, two species of Chrysomelinae, two species of Bruchinae and four species of Galerucinae. The phylogenetic trees were constructed with MEGA 7 (Kumar et al. 2008) using maximum-likelihood method. The phylogenetic position of *P. champaka* was closely clustered with five other Cassidinae species (Figure 1), which was consistent with the traditional morphological classification within Cassidinae.

**Figure 1.**
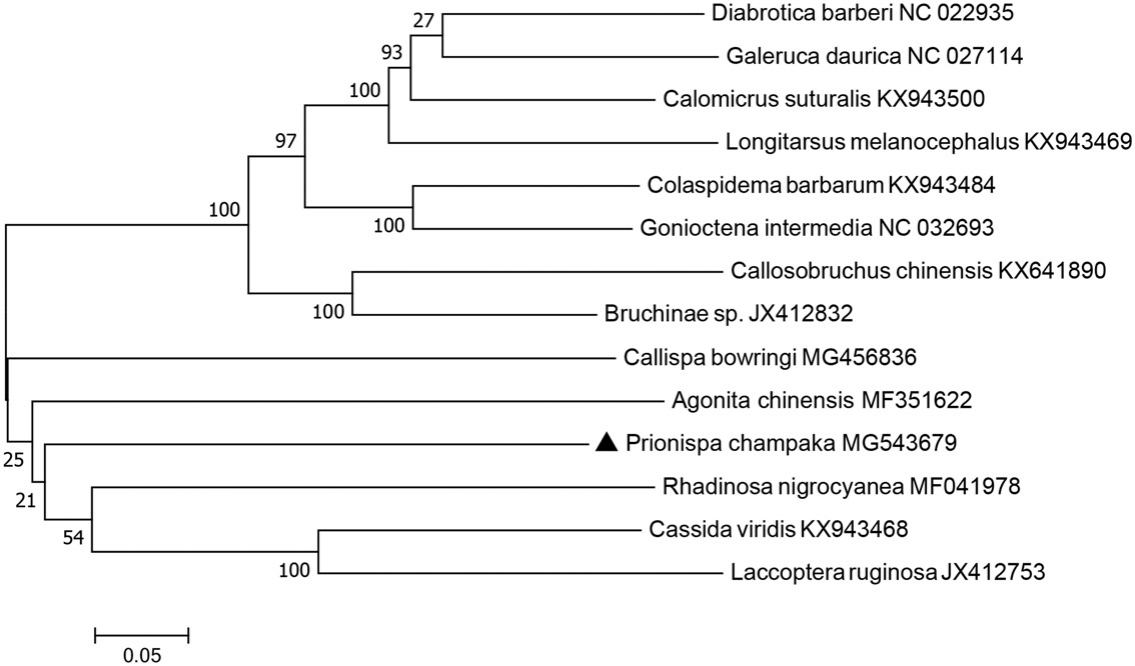
Maximum-likelihood tree indicating evolutionary relationships among *Prionispa champaka* and 13 other chrysomelid species based on mitochondrial PCGs concatenated dataset.

## References

[CIT0001] Bae JS, Kim I, Sohn HD, Jin BR. 2004 The mitochondrial genome of the firefly, *Pyrocoelia rufa*: complete DNA sequence, genome organization, and phylogenetic analysis with other insects. Mol Phylogenet Evol. 32:978–985. 15288070 10.1016/j.ympev.2004.03.009

[CIT0002] Cameron SL. 2014 Insect mitochondrial genomics: implications for evolution and phylogeny. Annu Rev Entomol. 59:95–117. 24160435 10.1146/annurev-ento-011613-162007

[CIT0003] Chen S, Yu P, Sun C, T’an C, Zia Y. 1986 Fauna Sinica (Insecta: Coleoptera: Hispidae). Beijing: Science Press. [In Chinese]

[CIT0004] Guo Q, Xu J, Dai X, Liao C, Long C. 2017a Complete mitochondrial genome of a leaf-mining beetle, *Rhadinosa nigrocyanea* (Coleoptera: Chrysomelidae) with phylogenetic consideration. Mitochondrial DNA Part B Resour. 2:446–448. 10.1080/23802359.2017.1357443 PMC7799965 33473857

[CIT0005] Guo Q, Xu J, Liao C, Dai X, Jiang X. 2017b Complete mitochondrial genome of a leaf-mining beetle, *Agonita chinensis* Weise (Coleoptera: Chrysomelidae). Mitochondrial DNA Part B Resour. 2:532–533. 10.1080/23802359.2017.1365650 PMC7800852 33473888

[CIT0006] Kumar S, Nei M, Dudley J, Tamura K. 2008 MEGA: a biologist-centric software for evolutionary analysis of DNA and protein sequences. Brief Bioinformatics. 9:299–306. 18417537 10.1093/bib/bbn017 PMC2562624

[CIT0007] Liao C, Liu P, Xu J, Staines CL, Dai X. 2018 Description of the last-instar larva and pupa of a leaf-mining hispine – *Prionispa champaka* Maulik, 1919 (Coleoptera, Chrysomelidae, Cassidinae, Oncocephalini). ZooKeys. 729:47–60. 10.3897/zookeys.729.21041 PMC5799732 29416391

[CIT0008] Nie R, Yang X. 2014 Research progress in mitochondrial genomes of Coleoptera. Acta Entomologica Sinica. 57:860–868. [In Chinese]

[CIT0009] Sayadi A, Immonen E, Tellgren-Roth C, Arnqvist G. 2017 The evolution of dark matter in the mitogenome of seed beetles. Genome Biol Evol. 9:2697–2706. 29048527 10.1093/gbe/evx205 PMC5737749

[CIT0010] Staines CL. 2015 Tribe Oncocephalini. Catalog of the hispines of the World (Coleoptera: Chrysomelidae: Cassidinae) [accessed 2017 Oct 1]. http://entomology.si.edu/collections_coleoptera-hispines.html.

[CIT0011] Wang Q, Tang G. 2017 Genomic and phylogenetic analysis of the complete mitochondrial DNA sequence of walnut leaf pest *Paleosepharia posticata* (Coleoptera: Chrysomeloidea). J Asia-Pac Entomol. 20:840–853.

